# Differentiating True Occlusion from Pseudo-Occlusion: The Role of Extended Multiphase Computed Tomography Angiography Scan Range in Internal Carotid Artery Occlusion

**DOI:** 10.3390/diagnostics15172265

**Published:** 2025-09-07

**Authors:** Hsin-Fan Chiang, Cheng-Chih Hsieh, Shih-Yang Wei, An-Bang Zeng, Ching-Chia Huang, Cheng-Han Chan, Chao-Yang Zheng, Chun-Chao Huang

**Affiliations:** 1 Department of Radiology, MacKay Memorial Hospital, Taipei 104217, Taiwan; chianghf99@gmail.com (H.-F.C.);; 2 Department of Medicine, MacKay Medical University, New Taipei City 252005, Taiwan; 3 MacKay Junior College of Medicine, Nursing, and Management, Taipei 112021, Taiwan

**Keywords:** ICA, pseudo-occlusion, acute ischemic stroke, mCTA, carotid bifurcation stenosis, endovascular thrombectomy

## Abstract

**Background**: Accurate localization of internal carotid artery (ICA) occlusion is critical for optimizing endovascular thrombectomy (EVT) strategies. Conventional multiphase CT angiography (mCTA) often omits the carotid bifurcation in delayed phases, limiting differentiation between true cervical ICA occlusion and pseudo-occlusion. **Methods**: We retrospectively analyzed 56 acute ischemic stroke patients with ICA occlusion who underwent EVT and extended-range mCTA between 2016 and 2020. The scan range of the second and third arterial phases was modified to include the carotid bifurcation. Imaging patterns were evaluated to distinguish bifurcation stenosis with superimposed occlusion from proximal ICA occlusion, and to infer thrombus location by comparing arterial opacification levels across phases. **Results**: Extended mCTA significantly improved visualization of ICA enhancement patterns in delayed phases (*p* < 0.001). Cases with bifurcation stenosis showed consistently lower and stable opacification levels across phases, whereas proximal ICA occlusion demonstrated progressive contrast advancement. Distal occlusion, particularly beyond the ophthalmic artery, showed higher opacification. Including the carotid bifurcation increased scan length by ~10%, with acceptable radiation exposure. **Conclusions**: Incorporating the carotid bifurcation into delayed mCTA phases enhances the ability to differentiate occlusion subtypes and estimate thrombus location. This refined imaging approach enables better EVT planning, including device selection and procedural timing, thereby improving patient outcomes in acute stroke care.

## 1. Introduction

Stroke is a leading cause of death and disability worldwide [1]. Endovascular thrombectomy (EVT) has become the standard of care for acute ischemic stroke caused by large vessel occlusion, as multiple randomized trials have demonstrated its superiority in improving functional outcomes [2,3,4,5,6]. Consequently, EVT is now included in treatment guidelines, while the substantial burden of post-stroke care on society and healthcare systems remains a major concern [7]. Early intervention and comprehensive stroke strategies are therefore essential to mitigate the high mortality and disability associated with stroke [8].

Multiphase computed tomography angiography (mCTA) has emerged as the imaging gold standard for detecting large vessel occlusion and assessing collateral circulation [9,10,11]. However, when focusing on internal carotid artery (ICA) occlusion, the presence of pseudo-occlusion—where distal ICA occlusion is misdiagnosed as proximal occlusion or carotid bifurcation stenosis with acute superimposed occlusion—poses significant challenges [12,13,14]. This misinterpretation can have critical implications for treatment planning, particularly in delineating the appropriate therapeutic approach based on the actual thrombus location. For instance, the presence of carotid bifurcation stenosis may necessitate percutaneous transluminal angioplasty (PTA) [15], while proximal ICA thrombi may require larger bore aspiration tubes, in contrast to distal occlusions that are better addressed using relatively smaller bore aspiration tubes. Consequently, the ability to identify ICA occlusion patterns via mCTA holds considerable promise in optimizing treatment strategies, allowing for the preparation of essential tools and the selection of the most suitable interventional devices [16].

Pattern-recognition signs in the proximal cervical ICA—such as a tapered “beak” configuration or a gradual decline in contrast—considerably improve the accuracy of identifying pseudo-occlusion [17]. However, the chief technical limitation of conventional multiphase CTA is its limited z-axis coverage: in the original protocol, the delayed arterial phases extend only from the skull base to the vertex, omitting the carotid bifurcation—precisely where distinguishing true cervical occlusion from distal pseudo-occlusion is most critical.

The present study directly targets these shortcomings. We modified the conventional three-phase protocol by extending the acquisition window of the second and third phases caudally to the carotid bifurcation. This “extended mCTA” is designed to differentiate pseudo-occlusion from true occlusions and achieve these goals with only a modest (~10%) increase in radiation dose. We hypothesize that extended mCTA will translate these refinements into actionable information for EVT operators—allowing tailored device selection, shorter procedural times, and ultimately better functional outcomes.

Accordingly, the two main purposes of this article are the following. Firstly, it seeks to examine the differences in imaging characteristics depicted by mCTA between the cases of severe ICA bifurcation stenosis with acute in situ occlusion and those without such stenosis presenting as pseudo-occlusion. Secondly, after excluding cases of severe carotid bifurcation stenosis with acute superimposed occlusion, this study aims to investigate whether there are differences in CTA image patterns when using different ICA segments as cut points to separate proximal and distal ICA occlusion, in order to infer the thrombus location. This research aims to contribute to a deeper understanding of mCTA’s role in optimizing treatment planning, facilitating the EVT procedure and thereby improving patient outcomes in the context of acute ischemic stroke with ICA occlusion.

## 2. Methods

This is a retrospective study to enroll cases of acute ischemic stroke with large vessel occlusion between 2016 and 2020. This study had been approved by the Institutional Review Boards and Ethics Committees of MacKay Memorial Hospital (ethics approval number: 20MMHIS361e). All methods performed in this study were carried out in accordance with the Declaration of Helsinki and the hospital’s and government guidelines and regulations. Due to the retrospective nature of this study, informed consent for each included patient was waived by the Institutional Review Board of MacKay Memorial Hospital. The inclusion criteria were (1) having multiphase CTA images, (2) receiving EVT, and (3) the final occlusion site at the ICA based on angiographic findings. Exclusion criteria were (1) presence of motion artifact on multiphase CTA images, (2) poor enhancement quality on multiphase CTA, defined as arterial attenuation <200 HU measured in the contralateral internal carotid artery (ICA) or basilar artery (BA), and (3) presence of reverse arterial loop at the cervical segment of ICA. Patients with severe motion artefact or sub-optimal contrast enhancement were excluded because such conditions markedly degrade the diagnostic accuracy of CTA for large vessel occlusion. Reverse-loop anatomy was excluded because it shortens the measured cranio-caudal distance, potentially biasing our key outcome. The occlusion segment of the ICA was defined by the angiographic result during EVT when the segment showed persistent flow arrest after repeated roadmap evaluation with step-by-step advance of the aspiration tube or at the segment where the aspiration tube sucked the clot. The ICA was divided into 7 segments: C1: cervical segment; C2: petrous segment; C3: lacerum segment; C4: cavernous segment; C5: clinoid segment; C6: ophthalmic segment; and C7: communicating segment. If the occluded location was at the carotid bifurcation, the level was recorded as C0.

### 2.1. Procedure of Multiphase CTA

Multiphase CTA in this study was based on the original design [16] but slightly modified on the scan range. A non-enhanced CT scan was performed from head to neck (from the upper part of the aortic arch to the vertex), an arterial phase CT scan from head to neck, an 8 s delayed arterial phase CT scan for the whole head, and a 16 s delayed arterial phase CT scan for the whole head. The scan ranges of the original design on the last two delayed arterial phases were both from the skull base to the vertex, but the modified scan ranges in this study were from the inferior margin of the mandible to the skull vertex because the coverage of carotid bifurcation on the two delayed arterial phases provided important imaging information to predict the occlusion site of ICA according to our past clinical experience, especially for identifying potential underlying carotid bifurcation stenosis with acute superimposed occlusion. The contrast injection rate was 4 mL/s from either left or right cubital fossa. The start of the scan of the first arterial phase was based on the bolus tracking at the aortic arch when the Hounsfield unit achieved 100 more than the baseline density. The raw images were 1.5 mm on a Siemens SOMATOM Definition AS CT Scanner (Siemens Healthcare GmbH, Erlangen, Germany) and 1 mm on a Canon Aquilion PRIME CT Scanner (Toshiba Medical Systems, Nasu, Japan). Reconstruction with the maximum intensity projection (MIP) technique was applied on the first arterial phase CT images to produce axial, coronal, and sagittal MIP images, as well as on the second and third arterial phase CT images to produce axial MIP images. The settings of MIP were 20 mm slice thickness with 10 mm increment for axial reconstructed MIP images and 10 mm slice thickness with 4 mm increment for coronal and sagittal reconstructed MIP images.

### 2.2. Multiphase CTA Measurement

**Figure 1:**
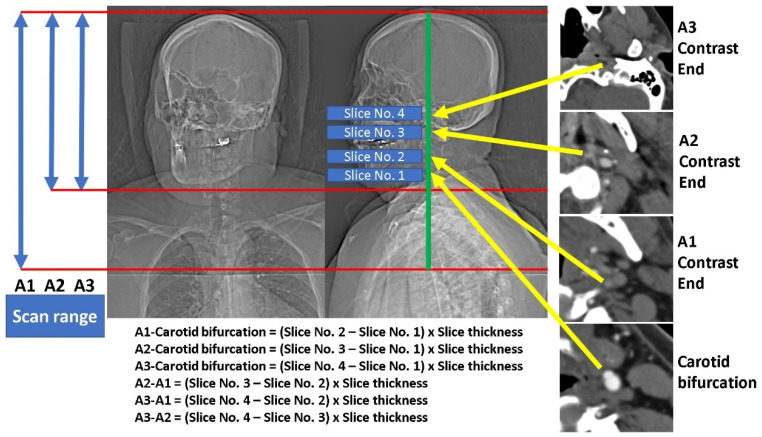
The demonstration of the length measurements of the highest enhancement level in the first (A1), second (A2), and third (A3) arterial phases from the carotid bifurcation and between each other.

In the enrolled subjects, all the multiphase CTA images of these subjects were reviewed, and the measurements were recorded by the consensus of two experienced neuroradiologists and one senior radiological technician. Total slices were recorded for each of the three arterial phases. The slice number of the first arterial phase, corresponding to the first slice of the two delayed arterial phases, was recorded. The slice numbers of the three arterial phases, corresponding to the disc levels of C2-3, C3-4, C4-5, C5-6, C6-7, C7-T1, and carotid bifurcation, inferior margin of the mandible, and skull base, were recorded. From the caudal to the cranial direction, the occluded ICA was checked slice-by-slice to record the first slice number when the contrast enhancement disappeared in each of the three arterial phases. In addition, whether there was presence of fluid-fluid level in the occluded ICA in any of the three arterial phases was recorded. The length measurement in this study is the product of the difference between the two target slice numbers and the thickness of the images. The measurements for each subject are depicted in Figure 1.

### 2.3. Statistical Analysis

The statistical analysis was performed using SPSS software, version 26.0 (IBM, Armonk, NY, USA). The visibility of the transitional zone of enhancement of ICA was evaluated between subjects with and without coverage of carotid bifurcation on the two delayed arterial phases. Presence of fluid-fluid level was used to differentiate occlusion level by different cut-off segments and to evaluate the association with the lengths between highest ICA enhancement level and carotid bifurcation on the three arterial phases and with the length differences of the highest ICA enhancement level among the three arterial phases. For the cases with C7 occlusion, the presence of posterior communicating artery (PCoA) was used to evaluate the association with the length differences of abovementioned highest ICA enhancement level. After excluding the C0 level occlusion, the abovementioned length differences were evaluated for normality by using the Kolmogorov–Smirnov test. Then, these length differences were used to differentiate occlusion level by different cut-off ICA segments. For cases with C0 to C5 occlusion levels, the same analytic processes were conducted to compare occlusion level at C0 and at C1-5 ICA segments. The total scan lengths were compared when the two delayed arterial phases started at the carotid bifurcation, at the inferior margin of the mandible, and at the skull base. The percentage of covering the carotid bifurcation was evaluated on the basis of using different landmarks as the start of the scan, including the inferior margin of the mandible and disc levels of C2-3, C3-4, C4-5, C5-6, C6-7, and C7-T1.

Descriptive statistics were used to evaluate demographic data. The *T*-test or the Mann–Whitney U test was used for continuous variables with or without normal distribution, respectively, and the Chi-square test with Fisher’s exact test was used for binary variables to compare between-group differences. All statistical tests were two-sided with the significance threshold set at 0.05.

## 3. Results

**Figure 2:**
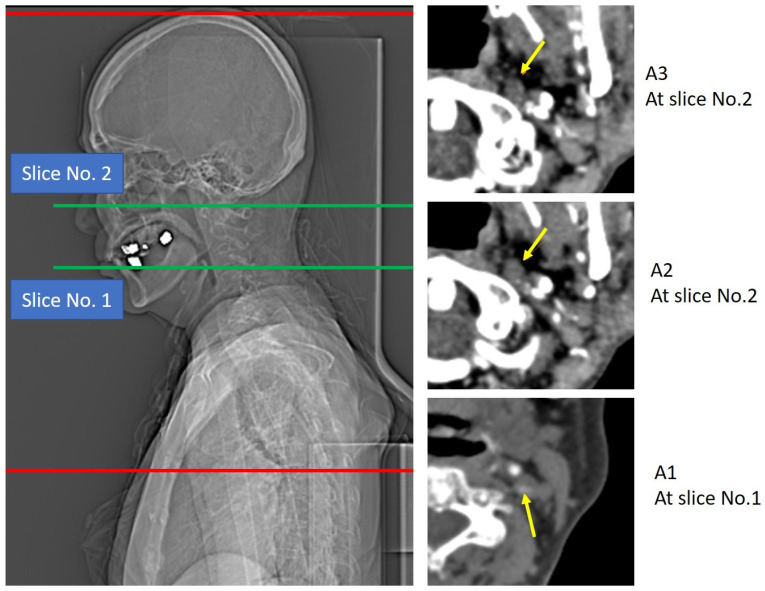
This case demonstrates the highest level of contrast enhancement at the proximal cervical segment of the left internal carotid artery during the first arterial phase (A1). In the second (A2) and third (A3) arterial phases, however, the limited z-axis coverage of the standard multiphase CTA, which does not extend to the carotid bifurcation, poses challenges for accurate image interpretation. The yellow arrows indicate the corresponding location of the ICA in A1, A2, and A3, respectively. Notably, Slice No. 2 corresponds to the skull base landmark routinely used to define the lowest z-axis level covered in the second and third arterial phases of the standard multiphase CTA protocol. This highlights the importance of adequate coverage to avoid misinterpretation of arterial occlusion or pseudo-occlusion.

There were 56 cases included in this study. The ICA occlusion level was 7 at C0, 4 at C1, 3 at C2, 1 at C3, 5 at C4, 13 at C5, 10 at C6, and 13 at C7. Of them, 33 cases had images of the two delayed arterial phases, including the location of carotid bifurcation, and the transitional zone of the ICA enhancement in the two delayed arterial phases was all visible in these 33 cases. Only in 7 of the remaining 23 cases was the transitional zone of the ICA enhancement detected in the two delayed arterial phases (Figure 2). The *p*-value was <0.001. In the 49 cases with ICA occlusion level at C1 to C7 segments, fluid-fluid level in the ICA was present in 3 C1 occlusion cases, 2 C2 occlusion cases, 1 C3 occlusion case, 5 C4 occlusion cases, 8 C5 occlusion cases, 8 C6 occlusion cases, and 8 C7 occlusion cases. No significant difference of occurrence of fluid-fluid level was found by using any cut-off level for ICA occlusion, with *p* values ranging from 0.297 to 1.000. Between cases of present and absent fluid-fluid level of ICA, the distributions of the following lengths were not significantly different: between the highest ICA enhancement level in the first arterial phase and carotid bifurcation (A1-Bifurcation length), between the highest ICA enhancement level in the second arterial phase and carotid bifurcation (A2-Bifurcation length), between the highest ICA enhancement level in the third arterial phase and carotid bifurcation (A3-Bifurcation length), between the highest ICA enhancement level in the first and second arterial phases (A2-A1 length), between the highest ICA enhancement level in the second and third arterial phases (A3-A2 length), and between the highest ICA enhancement level in the first and third arterial phases (A3-A1 length). The *p*-values ranged from 0.201 to 0.821. In the 13 cases with C7 occlusion level, between cases of present and absent PCoA, the distributions of the abovementioned six lengths were statistically different in A3-A1 length (*p* = 0.030) but similar in A1-Bifurcation length (*p* = 0.093), A2-Bifurcation length (*p* = 0.755), A3-Bifurcation length (*p* = 0.530), A2-A1 length (*p* = 0.073), and A3-A2 length (*p* = 0.106). In the 49 cases with ICA occlusion level at C1 to C7 segments, the distributions of the six lengths using different cut-off occlusion level showed statistically significant differences in A1-Bifurcation length (*p* = 0.013), A2-Bifurcation length (*p* = 0.011), and A3-Bifurcation length (*p* = 0.004) between C1-2 occlusion cases and C3-7 occlusion cases; in A1-Bifurcation length (*p* = 0.007), A2-Bifurcation length (*p* = 0.015), and A3-Bifurcation length (*p* = 0.005) between C1-3 occlusion cases and C4-7 occlusion cases; in A1-Bifurcation length (*p* < 0.001), A2-Bifurcation length (*p* = 0.002), and A3-Bifurcation length (*p* = 0.004) between C1-4 occlusion cases and C5-7 occlusion cases; in A1-Bifurcation length (*p* = 0.002), A2-Bifurcation length (*p* = 0.020), and A3-Bifurcation length (*p* = 0.011) between C1-5 occlusion cases and C6-7 occlusion cases; and in A1-Bifurcation length (*p* = 0.006) between C1-6 occlusion cases and C7 occlusion cases (Table 1). In the 33 cases with C0 to C5 occlusion level, between cases with C0 and C1-5 occlusion levels, the distributions of the six lengths were similar for A3-A2 length (*p* = 0.262) but statistically different in A1-Bifurcation length (*p* = 0.018), A2-Bifurcation length (*p* = 0.003), A3-Bifurcation length (*p* = 0.002), A2-A1 length (*p* = 0.002), and A3-A1 length (*p* = 0.005) (Table 2).

The mean total scan length of all cases was expected to be 1005.71 ± 106.44 mm when the two delayed arterial phases were scanned from the skull base, 1113.47 ± 112.00 mm when the two delayed arterial phases were scanned from the carotid bifurcation, and 1156.04 ± 105.75 mm when the two delayed arterial phases were scanned from the inferior margin of the mandible. The results of the pair T test for these three means of total scan length were significantly different with a *p* value < 0.001. The percentages of covering the carotid bifurcation using different landmarks as the start of the scan were 85.71% using the inferior margin of the mandible, and 1.79%, 56.57%, 91.07%, 100%, 100%, and 100% using disc levels of C2-3, C3-4, C4-5, C5-6, C6-7, and C7-T1, respectively.

## 4. Discussion

In this study, we found that the highest contrast opacification in ICA from the carotid bifurcation in all three arterial phases was significantly different between carotid bifurcation stenosis with in situ occlusion and ICA occlusion at C1 to C5 segments, especially in A2 and A3 arterial phases. The separation of these two distinct occlusion patterns relied on the capture of ICA enhancement in the A2 and A3 arterial phases, but the traditional scan setting from the skull base demonstrated as high as 69.6% possibility of inability to detect contrast opacification of ICA in these two arterial phases.

Upon excluding cases of carotid bifurcation stenosis with acute superimposed occlusion, when using the ophthalmic artery as the cut-point to define proximal and distal ICA occlusion, the distance of arterial opacification from the carotid bifurcation in all three arterial phases was consistently greater in distal ICA occlusion. However, in cases of C7 segment occlusion, between the presence or not of the PCoA, the significant difference in ICA opacification distance was only observed in the A1 phase.

When comparing between carotid bifurcation stenosis with acute superimposed occlusion and proximal ICA occlusion (C1-5 segments), there were observed differences in the distance of the highest ICA contrast enhancement from the bifurcation across A1, A2, and A3 phases. Notably, the *p*-values for A2 and A3 were lower than for A1, suggesting a more significant difference regarding contrast enhancement in the delayed phases. Furthermore, there were statistical differences between the intervals of distance differences A2-A1 (the highest ICA enhancement level in A2 minus that in A1) and A3-A1 (the highest ICA enhancement level in A3 minus that in A1). Theoretically, in the case of carotid bifurcation stenosis with acute superimposed occlusion, the opacification within the ICA would stop at the site of stenosis in all three arterial phases. However, in proximal ICA occlusion at the C1-5 segments, there is a potential for the contrast to gradually progress distally in the ICA across the three arterial phases, which explains the findings of our study. Observing the height of ICA opacification and the differences between phases can assist in determining whether an occlusion is due to carotid bifurcation stenosis with acute superimposed occlusion. In such cases, when performing endovascular thrombectomy, it is essential to employ balloon angioplasty techniques early on, as reliance on aspiration or stent retriever alone is often ineffective. Traditional mCTA protocols that commence scanning from the skull base without encompassing the carotid bifurcation pose a significant limitation, with an estimated 69.6% probability of not visualizing the ICA opacification height in the A2 and A3 phases, compromising the ability to localize true occlusion site on mCTA. An extended scan range that includes the carotid bifurcation in the second and third arterial phases could resolve this issue, allowing for superior preoperative imaging to determine the presence of carotid bifurcation stenosis with acute superimposed occlusion and thereby facilitating the procedure of EVT.

After excluding cases of carotid bifurcation stenosis with acute superimposed occlusion, it was noted that distal occlusions generally demonstrated a higher level of ICA opacification in the A1, A2, and A3 phases as compared to proximal occlusions. However, when the cut-point of distal versus proximal occlusion was defined by the location of the PCoA, a significant difference in opacification was only evident in the A1 phase. Possible explanation for these observations is because the rate of pre-occlusive blood flow is related to the presence of arterial branches at the patent arterial segments, which can facilitate collateral circulation. The ICA begins to exhibit small branches from the C2 segment and more branches from the C4 segment. However, the most prominent and largest first arterial branch is the ophthalmic artery [18]. Our results suggest that when the C6 segment, which is after the orifice of the ophthalmic artery, is categorized as part of a distal occlusion, there is a consistently higher opacification level across all phases compared to proximal occlusions. Once the C6 segment is included in the proximal occlusion category, this significantly elevates the mean height of blood flow progression in the proximal group, reducing the differences in the A2 and A3 phases to be statistically insignificant. Furthermore, among the subset of C7 segment occlusions, when groups were differentiated based on the visibility of the PCoA on angiograms, the A3-A1 values were smaller in the group with a visible PCoA compared to those without. This suggests that while the PCoA is a relatively significant collateral vessel of the ICA, the absence of a PCoA may result in a slower flow rate than when it is present. However, since the C7 occlusion group already benefits from collateral flow via the ophthalmic artery, the overall flow rate is significantly enhanced regardless. Additionally, the flow through the PCoA sometimes competes with the posterior circulation, which may not reliably facilitate rapid unidirectional flow, possibly leading to a less pronounced impact on flow enhancement between groups with and without a PCoA. Consequently, our results show a group difference only in the A3-A1 length, which is with a longer temporal gap and can detect a subtle difference. In the presence of a PCoA, the smaller A3-A1 values indicate that the A1 phase already achieves a high level of ICA opacification, thereby limiting the potential for further increase by the A3 phase. Conversely, in the absence of a PCoA, the overall slower ICA flow rate allows for a greater distance of opacification progression over the extended time frame between the A3 and A1 phases. Additionally, we observed the occurrence of fluid-fluid levels associated with slowed flow but found no evidence that the presence of fluid-fluid levels could differentiate between proximal or distal occlusions, regardless of which ICA segment was used as the cut-point. In brief, the presence of collateral branches before an ICA occlusion point significantly affects the flow rate, resulting in variations in the height of ICA opacification, while the fluid-fluid level plays no role in defining thrombus location. The most substantial factor is whether the ophthalmic artery is situated before the occlusion point, allowing for collateral flow. Additionally, the presence or absence of the PCoA may slightly enhance blood flow. Hence, when the A1 phase of mCTA demonstrates a higher level of ICA opacification, it may be inferred that the occlusion is likely situated beyond the ophthalmic artery.

We used the total scan length as a proxy for radiation dosage and categorized the scans into three groups: traditional multiphase CTA, carotid bifurcation, and mandibular base. The non-enhanced and A1 phase images span from the aortic arch to the vertex in all groups, while A2 and A3 phases have varying starting points, depending on the group, to the same endpoint of the vertex. The traditional group begins at the skull base, the carotid bifurcation group starts at the carotid bifurcation, and the mandibular base group uses the inferior margin of the mandible as the initial point for scanning. There was a statistically significant difference in the total scan lengths among these groups, with the longest one being the mandibular base group, followed by the carotid bifurcation group and then by the traditional group. To include the carotid bifurcation in the A2 and A3 phases for a more accurate assessment of the ICA occlusion site, the total scan length would need to be extended by an average of 10%, which corresponds to approximately a 10% increase in radiation dosage. However, overall, this increase remains within the acceptable range of radiation exposure, and the benefit for patients outweighs the mild increase in radiation dose.

To capture the most precise location during a mCTA scan, a non-enhanced CT can be used as a reference to directly locate the carotid bifurcation, though this approach may require additional time and training for radiologic technologists. Identifying the carotid bifurcation on non-enhanced images is not necessarily straightforward and could significantly increase the time spent, thus potentially delaying subsequent EVT due to prolonged patient positioning on the CT table. Alternatively, using tomographic results to determine the starting points for A2 and A3 scans could be a faster and more convenient method for technologists. We evaluated different disc levels and the mandibular base as potential starting points and found that using a level below the C5-6 disc could cover the carotid bifurcation for all patients, while starting at the mandibular base included the carotid bifurcation in 85.71% of cases. Although using the disc level at C5-6 offers a higher inclusion rate for the carotid bifurcation, calculating the disc level on a tomogram is sometimes counterintuitive and time-consuming. Furthermore, patients with thicker shoulder soft tissue may obscure the level of C5-6 disc, and those who cannot maintain a neutral head position might present challenges in determining the disc level. In contrast, the mandibular base is a readily identifiable landmark and offers an 85.7% inclusion rate for carotid bifurcation, potentially making it a more practical choice for clinical practice.

There are some limitations in this study. This was a retrospective study with potential uncontrolled factors. The total enrolled subjects were limited, and minor changes might be obscured. Some measurements were a bit subjective, but we used consensus to define the final results. To simplify the measurement of ICA opacification, we used the difference between slice numbers of the images, which did not represent the true length of ICA, and we had to exclude those with reverse loop of the cervical segment of ICA because there might be a decreased slice number of distal ICA opacifications on the later arterial phase due to this anatomical problem. In addition, anatomical variations may influence the visualization of the carotid bifurcation, and patients with restricted neck mobility may present technical challenges during image acquisition.

## 5. Conclusions

In conclusion, extending the scan ranges in the A2 and A3 phases to include the carotid bifurcation can significantly enhance the diagnostic capability of mCTA in differentiating patients with carotid bifurcation stenosis with acute superimposed occlusion from those with ICA pseudo-occlusion. This can be practically achieved by using the inferior margin of the mandible as a landmark for topogram positioning, which provides a simple and effective method for ensuring the inclusion of this critical anatomical region.

In clinical practice, our findings suggest a practical imaging-based workflow for acute ischemic stroke patients with suspected ICA occlusion. When mCTA demonstrates abrupt cessation of ICA opacification at the carotid bifurcation across all phases, it strongly suggests underlying carotid bifurcation stenosis with superimposed occlusion, indicating the need for early consideration of angioplasty or stenting during EVT. Conversely, cases showing progressive contrast opacification across phases likely represent distal ICA occlusion, where aspiration or stent retriever techniques alone may be used.

Furthermore, more distal opacification of the ICA is suggestive of distal ICA occlusion, which is particularly useful for identifying occlusion sites located before or after the orifice of the ophthalmic artery. In clinical practice, cases demonstrating rapid dynamic contrast flows across the three mCTA phases or on angiography often indicate that the occlusion site is located distal to the ophthalmic artery. In contrast, relatively slow flow progression suggests that the occlusion site lies proximal to the ophthalmic artery. Our findings underscore the clinical value of extending the mCTA scan range and performing detailed preoperative assessment, offering the potential to tailor therapeutic strategies more effectively and to optimize patient care.

## Figures and Tables

**Table 1:** Differences in highest enhancement level by using different cut-points of occlusion level to compare proximal and distal occlusion in the 49 cases of ICA occlusion from C1 to C7.

	A1-CB	A2-CB	A3-CB	A2-A1	A3-A1	A3-A2
C1 vs.	10.38 ± 10.24	19.50	19.50	15.00	15.00	0.00
C2-7 (mm)	25.90 ± 26.19	49.20 ± 34.41	55.10 ± 33.81	22.21 ± 22.03	28.11 ± 24.91	5.90 ± 10.40
(N)	(4:45)	(1:35)	(1:35)	(1:35)	(1:35)	(1:35)
(*p*-value)	(0.231)	(0.611)	(0.389)	(0.889)	(0.722)	(0.556)
C1-2 vs.	8.14 ± 7.77	14.00 ± 4.82	15.00 ± 3.97	9.00 ± 5.20	10.00 ± 4.58	1.00 ± 1.73
C3-7 (mm)	27.38 ± 26.50	51.50 ± 34.09	57.67 ± 33.10	23.20 ± 22.33	29.36 ± 25.12	6.17 ± 10.65
(N)	(7:42)	(3:33)	(3:33)	(3:33)	(3:33)	(3:33)
(*p*-value)	(0.013 *)	(0.011 *)	(0.004 *)	(0.292)	(0.220)	(0.512)
C1-3 vs.	8.06 ± 7.20	16.50 ± 6.36	18.00 ± 6.82	10.88 ± 5.66	12.38 ± 6.05	1.50 ± 1.73
C4-7 (mm)	27.87 ± 26.64	52.36 ± 34.27	58.63 ± 33.16	23.41 ± 22.65	29.67 ± 25.45	6.27 ± 10.81
(N)	(8:41)	(4:32)	(4:32)	(4:32)	(4:32)	(4:32)
(*p*-value)	(0.007 *)	(0.015 *)	(0.005 *)	(0.421)	(0.269)	(0.716)
C1-4 vs.	7.62 ± 5.75	18.29 ± 6.25	23.21 ± 9.98	12.93 ± 5.83	17.86 ± 9.69	4.93 ± 5.25
C5-7 (mm)	30.78 ± 27.18	55.64 ± 34.33	61.57 ± 33.38	24.21 ± 23.63	30.14 ± 26.62	5.93 ± 11.25
(N)	(13:36)	(7:29)	(7:29)	(7:29)	(7:29)	(7:29)
(*p*-value)	(<0.001 *)	(0.002 *)	(0.004 *)	(0.611)	(0.480)	(0.456)
C1-5 vs.	15.19 ± 17.74	34.08 ± 24.65	39.86 ± 27.54	18.97 ± 13.81	24.75 ± 19.55	5.78 ± 7.75
C6-7 (mm)	35.30 ± 29.06	62.67 ± 37.12	68.36 ± 34.21	25.06 ± 27.64	30.75 ± 29.14	5.69 ± 12.58
(N)	(26:23)	(18:18)	(18:18)	(18:18)	(18:18)	(18:18)
(*p*-value)	(0.002 *)	(0.020 *)	(0.011 *)	(0.767)	(0.938)	(0.406)
C1-6 vs.	18.79 ± 21.26	40.90 ± 31.03	46.90 ± 31.97	21.15 ± 17.98	27.15 ± 21.57	6.00 ± 8.05
C7 (mm)	40.81 ± 30.18	63.33 ± 36.83	68.54 ± 34.16	23.75 ± 28.71	28.96 ± 30.95	5.21 ± 14.19
(N)	(36:13)	(24:12)	(24:12)	(24:12)	(24:12)	(24:12)
(*p*-value)	(0.006 *)	(0.112)	(0.067)	(0.518)	(0.704)	(0.265)

Abbreviations: A1, A2, and A3: first, second, and third arterial phases; C1, C2, C3, C4, C5, C6, and C7: seven segments of internal carotid artery; CB: carotid bifurcation; ICA: internal carotid artery; N: case number. *: *p*-value < 0.05.

**Table 2:** Differences in highest enhancement level between carotid bifurcation occlusion and proximal ICA occlusion from C1 to C5 levels.

	C0 (N = 7)	C1-5 (N = 26)	*p*-Value
A1-CB (mm)	2.64 ± 10.15	15.19 ± 17.74	0.018 *
	C0 (N = 4)	C1-5 (N = 18)	*p*-Value
A2-CB (mm)	4.13 ± 14.41	34.08 ± 24.65	0.003 *
A3-CB (mm)	5.52 ± 14.77	39.86 ± 27.54	0.002 *
A2-A1 (mm)	1.50 ± 1.22	18.97 ± 13.81	0.002 *
A3-A1 (mm)	2.63 ± 1.44	24.75 ± 19.55	0.005 *
A3-A2 (mm)	1.13 ± 2.25	5.78 ± 7.75	0.262

Abbreviations: A1, A2, and A3: first, second, and third arterial phases; C0: carotid bifurcation occlusion; C1, C2, C3, C4, and C5: proximal five segments of internal carotid artery; CB: carotid bifurcation; ICA: internal carotid artery; N: case number. *: *p*-value < 0.05.

## Data Availability

The datasets used and/or analyzed during the current study are available from the corresponding authors upon reasonable request.

## Abbreviations

A1: first arterial phase; A2: second arterial phase; A3: third arterial phase; CB: carotid bifurcation; C0: carotid bifurcation occlusion; C1–C7: cervical, petrous, lacerum, cavernous, clinoid, ophthalmic, and communicating ICA segments; CT: computed tomography; CTA: computed tomography angiography; EVT: endovascular thrombectomy; ICA: internal carotid artery; mCTA: multiphase computed tomography angiography; MIP: maximum intensity projection; PCoA: posterior communicating artery; PTA: percutaneous transluminal angioplasty; tPA: tissue plasminogen activator.
